# Optimisation of a Generic Ionic Model of Cardiac Myocyte Electrical Activity

**DOI:** 10.1155/2013/706195

**Published:** 2013-05-02

**Authors:** Tianruo Guo, Amr Al Abed, Nigel H. Lovell, Socrates Dokos

**Affiliations:** Graduate School of Biomedical Engineering, University of New South Wales, Sydney, NSW 2052, Australia

## Abstract

A generic cardiomyocyte ionic model, whose complexity lies between a simple phenomenological formulation and a biophysically detailed ionic membrane current description, is presented. The model provides a user-defined number of ionic currents, employing two-gate Hodgkin-Huxley type kinetics. Its generic nature allows accurate reconstruction of action potential waveforms recorded experimentally from a range of cardiac myocytes. Using a multiobjective optimisation approach, the generic ionic model was optimised to accurately reproduce multiple action potential waveforms recorded from central and peripheral sinoatrial nodes and right atrial and left atrial myocytes from rabbit cardiac tissue preparations, under different electrical stimulus protocols and pharmacological conditions. When fitted simultaneously to multiple datasets, the time course of several physiologically realistic ionic currents could be reconstructed. Model behaviours tend to be well identified when extra experimental information is incorporated into the optimisation.

## 1. Introduction

 The electrical activity of cardiac myocytes, including action potential (AP) waveforms and underlying membrane currents, has been extensively studied using a combination of microelectrode recording and mathematical modelling techniques [1]. The latter in particular has been utilised to provide a more quantitative and integrative understanding of the underlying mechanisms of cardiac electric activity. Biophysically detailed ionic models of cardiac cell electrophysiology are able to accurately reproduce a large range of behaviours, including membrane potential waveforms, specific ionic currents under voltage-clamp protocols, AP membrane current dynamics, and Ca^2+^ alternans [2, 3]. However, the ever-increasing number of variables in such models renders them computationally expensive when integrated into higher-dimensional tissue or whole heart simulations, limiting their utility.

As an alternative, simplified phenomenological models have been widely utilised in electrophysiological simulations due to their minimal complexity and computational cost. The first such generic model was the Fitzhugh-Nagumo (FHN) formulation published in 1961 [4], simplifying the four-variable Hodgkin-Huxley (HH) nerve axon model [5] into a two-variable formulation by eliminating gating variables with rapid time constants. Since its publication, FHN-type models have been frequently used in multicellular tissue simulations [6–9]. In 1998, Fenton and Karma published an improved phenomenological model based on the biophysically detailed Luo and Rudy [10] and Beeler and Reuter [11] ventricular cell ionic models in order to simulate ventricular fibrillation [12]. Given appropriate parameters, the ability of the three-variable Fenton-Karma model to reproduce AP duration restitution properties was shown to be comparable to biophysically detailed models [13]. By introducing an additional variable to this model, the Fenton-Cherry modification was also able to reproduce AP waveforms from pulmonary vein and left atrial myocytes [14]. Despite the successful application of these phenomenological models, their inherent oversimplicity may restrict their utility, particularly when simulating complex phenomena such as electrical remodelling during sustained arrhythmia [15] or the effects of selective ionic channel blockers [16]. For example, it is doubtful whether such models can accurately reproduce the range of AP waveforms recorded from the same myocyte under various electrical pacing frequencies or multiple degrees of selective ion channel block by drugs, due to the small number of membrane currents in these models.

Another significant challenge in cardiac single cell ionic modelling lies in estimating the host of parameters governing the kinetics and densities of the various membrane ion channels, pumps, and exchangers, as well as parameters governing intracellular ionic cycling and buffering mechanisms, all from readily available experimental data obtained in a given myocyte. Given enough parameters, ionic models may only be able to accurately reproduce ionic mechanisms under the precise experimental conditions they were fitted to in the first place. Models with modified parameters to fit another set of experimental data may lose their original mechanisms [3, 17], limiting their predictive utility. Moreover, the ever-increasing complexity of such models makes parameter estimation a highly difficult and time-consuming task. Although previous studies have undertaken parameter optimisation in cardiac ionic models [18, 19], these have all used a relatively limited subset of parameters (typically maximum membrane conductances only) to fit AP records. Until now, a very limited number of optimisation algorithms have been successfully used for large-scale optimisation of cardiac cell ionic models.

 In response to these challenges, we have formulated a simplified HH-type generic model to accurately reconstruct a range of AP waveforms recorded from tissue-intact myocytes in rabbit sinoatrial (SAN) and right and left atrial (RA, LA) tissue preparations. The model structure is flexible and modular and the optimised models are able to reproduce complex behaviour such as the change in AP morphology due to selective ion channel block or high-frequency paced stimulation. Model parameters were estimated using a computationally simple, multiobjective AP optimisation approach based on a custom curvilinear-gradient method [20]. The improvement in membrane current reconstruction gained by optimising the model to simultaneously fit different AP experimental waveforms was examined.

## 2. Methods

### 2.1. Generic Cell Model Formulations

 The generic form of the model is given by

(1) $$\begin{array}{c} \frac{dE_{m}}{dt}\frac{dE_{m}}{dt}=-\frac{1}{C_{m}}\left(i_{\text{stim}}+i_{L}+\sum_{j=1}^{N}i_{j}\right), \end{array}$$

where *E*_*m*_ (mV) denotes the transmembrane potential, *t*(*s*) denotes time, *C*_*m*_ (*μ*F/cm^2^) is the specific membrane capacitance, *i*_*L*_ (nA/cm^2^) refers to the time-independent (leakage) current, *i*_stim_ (nA/cm^2^) is the applied stimulus, *i*_*j*_ (nA/cm^2^) denotes the *j*th time-dependent ionic current density, and *N* is the user-defined number of time-dependent currents, the value of which depends on the complexity of the data to which the model is to be fitted. The ability to set the number of currents makes the model generic and modular. The time-dependent currents are represented by a two-gate HH scheme:

(2) $$\begin{array}{c} i_{j}={\overset{-}{g}}_{j}p_{j}q_{j}\left(E_{m}-E_{\text{rev},j}\right), \end{array}$$

where ${\overset{-}{g}}_{j}$ (*μ*S/cm^2^) is the maximum ion channel conductance, *p*_*j*_ and *q*_*j*_ are dimensionless gating variables, and *E*_rev,j_ (mV) is the reversal potential for membrane current *j*. The time-independent leakage current is described by

(3) $$\begin{array}{c} i_{L}={\overset{-}{g}}_{L}\left(E_{m}-E_{\text{rev},L}\right), \end{array}$$

where ${\overset{-}{g}}_{L}$ (*μ*S/cm^2^) and *E*_rev,L_ (mV) are the maximum ion channel conductance and reversal potential, respectively. For the gating variables in (2), HH kinetics [5] are employed to specify first order differential equations (ODEs):

(4) dpjdt=αp,j(1−pj)−βp,jpj,dqjdt=αq,j(1−qj)−βq,jqj,

where *α*_*p*,*j*_, *β*_*p*,*j*_, *α*_*q*,*j*_, and *β*_*q*,*j*_ (s^−1^) represent opening and closing rates for the corresponding gating variable. These rates are given by sigmoidal functions of the membrane potential *E*_*m*_:

(5) $$\begin{array}{c} \alpha ,\beta =\frac{k}{1+\exp\left[s\left(E_{m}-E_{50}\right)\right]}, \end{array}$$

where *k* (s^−1^), *s* (mV^−1^), and *E*_50_ (mV) are parameters specific to each rate, with each *E*_50_ parameter value shared between the *α* and *β* pair for each gating variable.

Therefore in total, the generic model contains 2*N* + 1 ODEs and 12*N* + 2 parameters. In principle, the higher the value of *N*, the more degrees of freedom and the more complex are the electrophysiological behaviours which can be reproduced, at the cost of decreased computational efficiency. 

### 2.2. Parameter Optimisation

 A custom curvilinear gradient-based least-squares optimisation method, combining the advantages of both Newton and steepest-descent methods [20], was carried out on a standard desktop PC using Matlab software (The Mathworks Inc., USA). The curvilinear gradient method may be briefly described as follows: we assume that an array of *m* × 1 data points **d** are to be fitted by an ODE system *f*(**p**), where **p** is an *n* × 1 array of parameters. The *m* × 1 residual array **r** between model and data is given by

(6) $$\begin{array}{c} r\left(p\right)=f-d. \end{array}$$

In general, the model output *f*(**p**) will be a nonlinear function of the parameter array **p**. However, the residual can be linearized using the approximation:

(7) $$\begin{array}{c} r\left(\Delta p\right)=r_{0}+\left(\frac{\partial f}{\partial p}\right)\cdot \Delta p\\ \;\;=r_{0}+J\cdot \Delta p, \end{array}$$

where Δ**p** is the *n* × 1 array of parameter increments relative to the current parameter set **p**, and **r**_0_ is the array of residuals. A least-squares scalar objective function, *Q*, to be minimised is given by

(8)   Q(Δp)=r(Δp)T·r(Δp)=r0Tr0+2ΔpTJTr0+ΔpTJTJΔp=Q0+ΔpTG+12ΔpTHΔp,

where T denotes the transpose, **J** is the *m* × *n* Jacobian matrix, Q_0_ = **r**_0_^T^ **r**_0_ is the current objective value, **G** = 2**J** ^T^ **r**_0_ denotes the *n* × 1 gradient of the objective at the current parameter point **p**, and **H** = 2**J** ^T^ **J** is the Hessian matrix. If the model is well determined in its parameters, **H** will be nonsingular at the global minimum for *Q*.

Equation (8) represents a quadratic form in the parameter increment vector Δ**p**. The exact minimum of this quadratic form will occur at

(9) $$\begin{array}{c} \Delta p=-H^{-1}G \end{array}$$

and represents a full Newton step to the minimum objective value. In practice, however, the least squares scalar objective given by (8) will not be accurate for large Δ**p**, where the linear approximation of the residual in (7) breaks down. When Δ**p** is sufficiently small, (8) will be accurate. We can however search for the minimum objective along a curvilinear trajectory through the *n*-dimensional parameter space given by the curve of steepest descent of the quadratic form (8). This trajectory is given by

(10) $$\begin{array}{c} L\left(\alpha \right)=\left(e^{-H\alpha }-I\right)H^{-1}G, \end{array}$$

where *α* is a parameter between 0 and infinity, with *α*  =  0 corresponding to the current parameter location **p** and *α*  =  inf⁡ corresponding to the full Newton step mentioned previously. When the objective minimum has been found along the trajectory, the quadratic form (8) is recomputed, and another search is performed along a new curvilinear path. The approach is iteratively carried out until the least square objective is locally minimised. Full details of the method are described in Dokos and Lovell [20] including random restarts and iterative reweighting to find the global least squares minimum. In addition, some of the experimental AP waveforms recorded in the present study exhibited stimulus artifacts which could confound the optimisation process. These were effectively removed by assigning a weight of zero to the residual between model and data in these regions.

When optimising the model to fit multiple datasets simultaneously, the residual in (6) was formed by appending together the residuals of the model and the corresponding individual datasets. As a result, the calculation of the Jacobian matrix **J** in (7) was slightly modified. Three cases can be considered.

- Multiple data (*R* datasets) fitted using the assumption that each dataset shares the same parameter values, for example, fitting multiple AP data recorded from the same cell in response to different pacing conditions. **J** will be of size *m* × *n*, where *m* is the total number of data points across all records and *n* is the number of optimised model parameters.
- Multiple data (*R* datasets) fitted using the assumption that each model uses a unique set of parameters to fit each experimental dataset. Data-specific parameters *x*_1_ to *x*_*R*_, each of size *n* × 1, are used for datasets 1 to *R*. This process is equivalent to performing multiple single dataset optimisations independently, and then **J** will be of size *m* × *nR*.
- Multiple data (*R* datasets) fitted using a combination of both shared and data-specific parameters. This is the case for the drug-specific and tissue-specific optimisation scenarios presented. For *S* data-specific parameters (i.e., *S* parameters unique to each dataset), **J** will be of size *m* ×[*n* + (*R* − 1) × *S*].

Compared with single dataset fitting, more computational resources are required for multiobjective optimisations due to the larger size of the Jacobian matrix, as well as the fact that more local minima are likely to be present in the objective parameter space. 

### 2.3. Cardiac Tissue Recordings

 New Zealand White rabbits (6–24 months old) were anesthetised with 5% isofluorane, and 1000 IU of heparin was administered intravenously. A thoracotomy was performed and the heart was rapidly excised and placed in cold cardioplegia solution. SAN-RA or LA appendage tissue preparations were dissected and placed in a recording chamber, superfused with Tyrode's solution and oxygenated with 95% O_2_ and 5% CO_2_ to maintain the pH at ~7.4. Intracellular APs were recorded using sharp glass microelectrodes (resistance 50–100 MΩ). Recordings were amplified (gain × 10) and filtered (low pass, cutoff 10 kHz) using an Axoclamp 2B amplifier (Axon Instruments, USA) and sampled at 20 kHz using a USB-6251 analog/digital converter (National Instruments, USA) and a custom build data acquisition software programmed in Labview (National Instruments, USA). For the LA experiments, the tissue was paced using a STG1002 stimulator (MultiChannel Systems, Germany). Monophasic suprathreshold pulses, 2 ms in duration, were delivered using Teflon coated bipolar stainless steel electrodes (125 *μ*m diameter). All experiments were conducted in accordance with Australian animal research guidelines and were approved by the University of New South Wales Animal Ethics and Care Committee.

## 3. Results

### 3.1. Results of Optimised Minimal Generic Model

**Figure 1:**
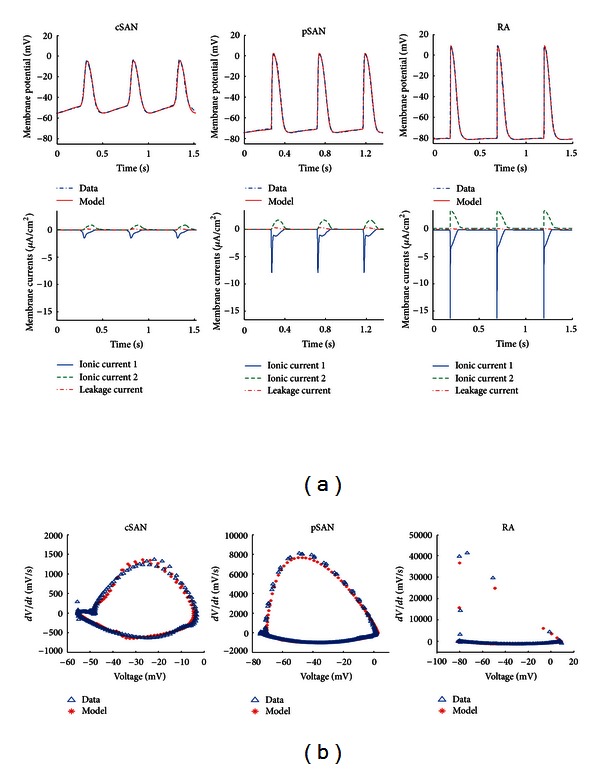
Optimisation based on single AP dataset. (a) Top panels: optimised model generated APs overlaid with experimental APs recorded from central sinoatrial node (cSAN), peripheral sinoatrial node (pSAN), and right atrial (RA) intact myocytes from rabbit sinoatrial tissue preparations. Lower panels: corresponding ionic and leakage currents generated by each cell model. (b) Phase plot (*dV*/*dt* versus *V*) of model APs overlaid with experimental data. The experimental voltage derivatives were obtained from first order finite differencing of the membrane voltage data.

 A minimal generic HH-type ionic model with two time-dependent membrane currents, one inward and one outward, in addition to a background leakage current was fitted to three consecutive spontaneous APs recorded from central sinoatrial node (cSAN), peripheral sinoatrial node (pSAN), and RA tissue-intact myocytes from rabbit sinoatrial tissue preparations. Optimised APs along with the corresponding ionic currents are shown in Figure 1. Lists of optimised parameter values and initial values for all model variables for each cell are given in Tables 1 and 2. In accordance with the data, the pSAN model-generated APs exhibited a faster upstroke, a more positive overshoot, and more negative maximum diastolic potential compared to the cSAN. Both types of SAN APs exhibited a slow depolarisation (pacemaker) phase which was absent in the RA cell, and, as a result, stimulus pulses of 2 ms duration and 30 *μ*A/cm^2^ amplitude were applied to trigger APs in the RA model. There was a gradual transition in the shape of the AP and ionic current waveforms from central SAN to atrial tissue. In particular, the transient spike of the inward current (ionic current 1) was nearly absent in the cSAN model and progressively increased both in magnitude and upstroke rate from pSAN to RA. The root mean square (RMS) error between the optimised models and data was 1.02 mV, 1.53 mV, and 1.69 mV for cSAN, pSAN, and RA myocytes, respectively.

**Figure 2:**
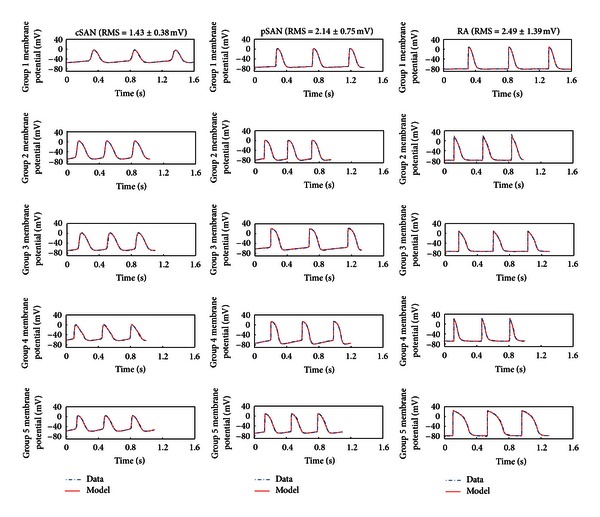
Generic model fits to a range of APs recorded from many myocytes. Optimised models overlaid with five recorded intact tissue APs from each of rabbit central SAN (cSAN), peripheral SAN (pSAN), and right atrial (RA) myocytes. Each single dataset was separately fitted with two time-dependent membrane currents and one leakage current. The average and standard deviation of the root mean square (RMS) error of the fits is also shown.

To assess the generalizability of the HH generic model and optimisation procedure in fitting a wide range of AP waveforms, five separate spontaneous AP recordings from each of the previous myocyte types were fitted using the three-current version of the generic model. Stimulus pulses of 2 ms duration and variable amplitude were used to excite the atrial models. The five sets of data comprised one group described previously (Group 1), plus an additional four sets (Groups 2–5) for each of the three myocyte types (cSAN, pSAN, and RA). Despite the inherent variation between APs recorded from the same myocyte type in different tissue preparations, the generic model was able to fit each dataset using only an inward, an outward, and one background membrane current (Figure 2). The RMS error for each cell type (*n* = 5, mean ± standard deviation) was 1.43 ± 0.38 mV (cSAN), 2.14 ± 0.75 mV (pSAN), and 2.49 ± 1.39 mV (RA). Compared with the fits shown in Figure 1 (Group 1), the mean RMS is marginally increased for all three cell types, likely due to the fact that unsmoothed experimental datasets (with mean peak-to-peak noise levels of ±0.96 mV) were used in fitting data Groups 3–5. Nonetheless, the model was able to reproduce the variability in AP waveform from the same cell type in different preparations.

### 3.2. Multiobjective Action Potential Optimisation 

#### 3.2.1. Multidataset Optimisation with Shared Parameters (Uniformly Paced Left Atrial Data)

**Figure 3:**
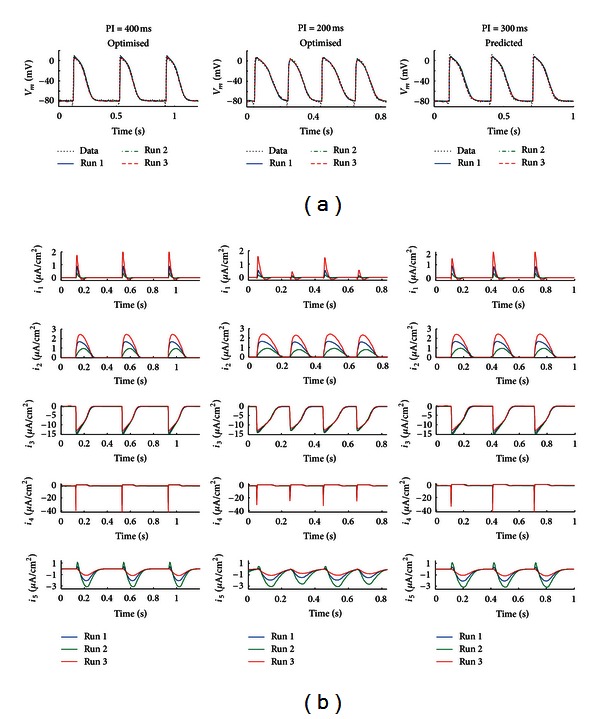
Multiple-dataset optimisation and validation of generic model to fit APs in response to uniform pacing at different frequencies. Experimental APs were obtained by pacing a left atrial intact myocyte at three different pacing intervals (PIs). Model optimisation was repeated three times (runs 1, 2, and 3), each starting at randomized initial parameter values. AP fits obtained for each run were very similar. (a) Three groups of optimised AP fits in response to pacing at intervals (PIs) of 400, 200, and 300 ms. The generic model with five time-dependent currents and one leakage current was simultaneously fitted to the first two datasets (PI = 400 ms and 200 ms) using a single set of model parameters. The optimised model was validated by its ability to reproduce AP responses to pacing at a PI of 300 ms, a dataset not used in the model optimisation. (b) Plots of model generated time-dependent currents for each pacing protocol for each optimisation run. Note the marked AP and membrane current beat-to-beat variations at PI = 200 ms.

 The generic model was also optimised to simultaneously fit two morphologically different LA AP waveforms from the same cell, recorded in response to stimulation at pacing intervals (PIs) of 400 ms and 200 ms (Figure 3(a)), using a single set of parameters. A total of five time-dependent ion currents and one leakage current were required to simultaneously fit the two sets of data, with results shown in Figure 3. Stimulus pulses of 2 ms duration and 13 *μ*A/cm^2^ in amplitude were used to elicit APs in the model. For a PI of 200 ms, the model generated AP characteristics and corresponding time-dependent ionic currents revealed beat-to-beat variations. Parameter values and initial values of all model variables are listed in Tables 3 and 4. The RMS errors between the optimised model and corresponding experimental data were 2.01 mV (PI = 400 ms) and 3.22 mV (PI = 200 ms). This optimised model was then used to predict the APs elicited at a PI of 300 ms, a dataset which was not used in the optimisation process. The additional experimental dataset can be reproduced with an RMS error of 2.46 mV (Figure 3, PI = 300 ms). 

 To test the reliability of the previous multiple-dataset based optimisation, two additional optimisation runs were carried out utilising the same LA paced data mentioned previously. Both optimisation runs started with the same stimulation settings, but used randomly generated initial parameter values. Iterations were terminated when the RMS error between model and experimental data reached a similar value to that obtained earlier. The resulting RMS errors between the optimised models and corresponding data were 2.01 and 2.40 mV (PI = 400 ms), 3.25 and 3.26 mV (PI = 200 ms), and 2.58 and 2.91 mV (PI = 300 ms) for optimisation runs 2 and 3, respectively.

Although model-generated APs from each optimisation run are almost identical, parameter values displayed significant variation between each run (see Tables 3 and 4 for a list of parameters and initial values for model parameters). The maximum relative difference (as a percentage of mean parameter value) is approximately 170% and the mean relative difference is 19%, indicating that model parameters cannot be uniquely identified, even when they are obtained from multiple dataset optimisations. Interestingly, despite the randomness of the initial parameter values for each optimisation run, the corresponding time-dependent ionic current waveforms are nearly identical and physiologically reasonable, except for differences in their scaling (Figure 3(b)). 

#### 3.2.2. Multidataset Optimisation with Shared Parameters (Randomly Paced Left Atrial Data)

**Figure 4:**
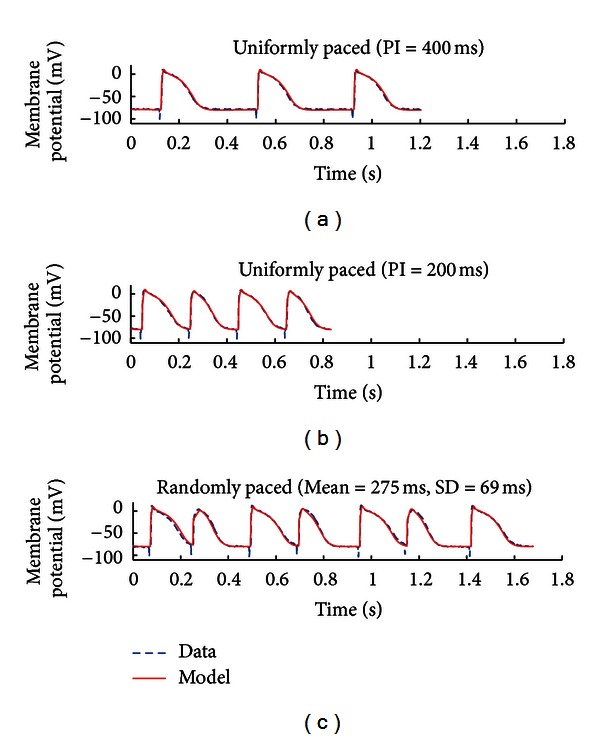
Multiple-dataset generic model fits to APs recorded in response to uniform and random pacing protocols. From top to bottom: AP fits in response to uniform pacing at intervals of 400 and 200 ms and a sequence of random pacing intervals (PIs). The random sequence was generated from a normal distribution of mean 275 ms and standard deviation 69 ms. The generic model with seven time-dependent currents and one leakage current was simultaneously fitted to all three experimental datasets using a single set of model parameters.

 A rabbit left atrial (LA) tissue preparation was electrically paced at uniform frequencies with a pacing interval (PI) of 200 ms and 400 ms, as well as randomly paced, and APs responses were recorded from the same myocyte (*n* = 1) for each pacing protocol. The pulse amplitude and duration were fixed for all protocols. A sequence of 100 random pulses was generated from a normal distribution of PIs, with mean and standard deviation of 275 ms and 69 ms, respectively. A sequence of seven pulses was selected and used in optimisation. In addition to AP alternans observed at a PI of 200 ms, more significant beat-beat variations in AP morphology were demonstrated in the randomly paced dataset, as shown in Figure 4 (lower panel). 

**Figure 5:**
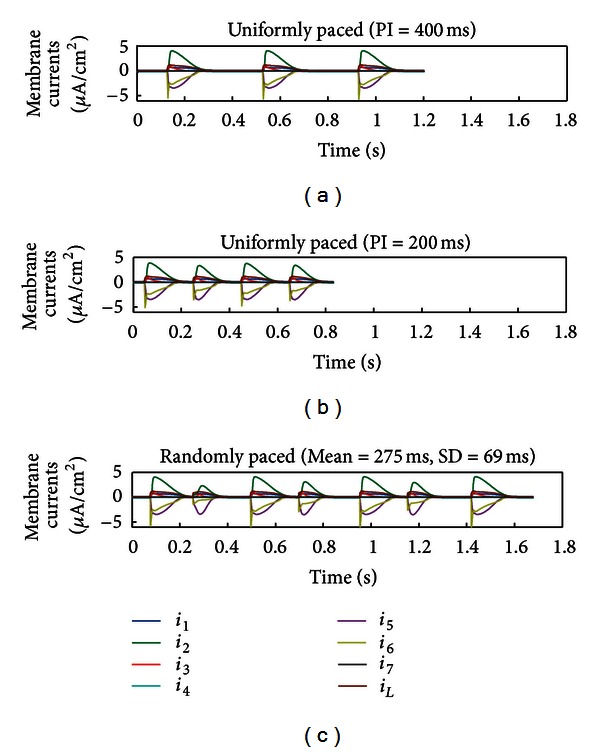
Reconstructed membrane currents of the optimised model in Figure 4. From top to bottom: membrane currents in response to stimulation at pacing intervals (PIs) of 400, 200 ms and a random sequence of PIs.

Using the generic ionic model, a total of seven time-dependent membrane currents and one leakage current were required to fit the multiple datasets simultaneously using a single set of parameter values (Table 5 lists the shared parameters), with distinct variable initial values for each dataset (Table 6). Stimulus pulses of 2 ms duration and 30 *μ*A/cm^2^ in amplitude were used to excite the model at the appropriate PI. The optimised model was able to accurately reproduce the experimental AP waveforms, even for the random-paced protocol (Figure 4). RMS errors between the optimised model and corresponding experimental data were 2.94 mV (PI = 400 ms), 3.51 mV (PI = 200 ms), and 3.91 mV (randomly paced).  Figure 5 illustrates the corresponding membrane currents generated by the optimised model when paced with the three protocols. Note that the generic model structure of Figures 4 and 5 is different from that of Figure 3 in respect of the total number of equations and parameters, since the number of time-dependent currents is not the same. The increase in the number of currents in the generic model of Figures 4 and 5 was necessitated by the requirement that the optimised model fit three AP datasets simultaneously, as opposed to the two AP datasets of Figure 3.

#### 3.2.3. Multidataset Optimisation with Combined Shared and Data-Specific Parameters

**Figure 6:**
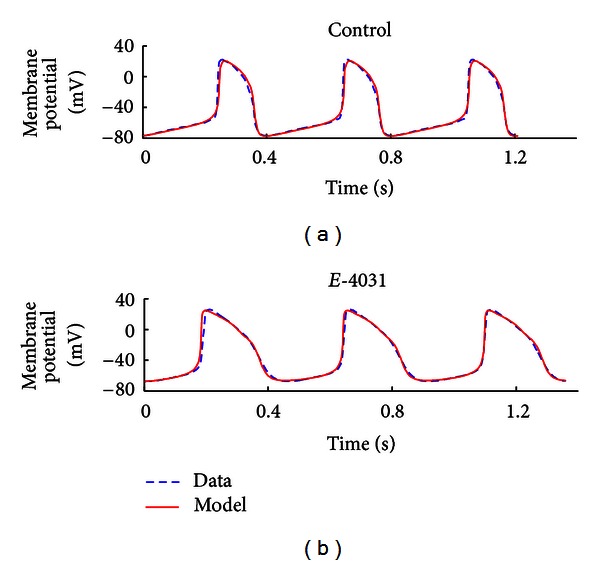
Drug-specific multiobjective optimisation. Top and bottom panels illustrate the optimised model (solid traces) overlaid with experimental AP data (dashed traces) of peripheral sinoatrial node (pSAN) APs under control conditions (a) and in the presence of 0.1 *μ*M E-4031, a selective blocker of *i*_Kr_ channels (b).

**Figure 7:**
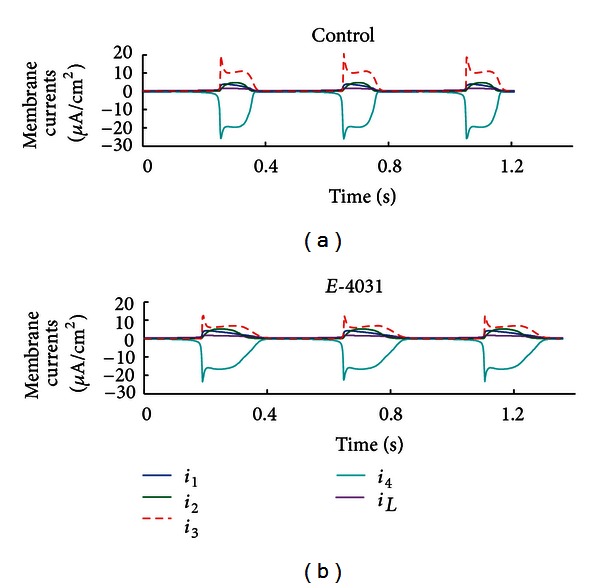
Reconstructed membrane currents for the drug-specific optimisation. Four time-dependent currents and one leakage current were included in the model. *i*_Kr_(*i*_3_), which was partially blocked by E-4031, is shown as a thick red dashed line.

*(i) Drug-Specific Case.* The generic ionic model was simultaneously fitted to two spontaneous peripheral sinoatrial node (pSAN) AP datasets from Kodama et al. [21]: a control set of APs and the AP response following the application of E-4031, a specific blocker of rapid delayed-rectifier potassium (*i*_Kr_) channels. To fit the generic model to both datasets, four time-dependent currents and one leakage current were required. Current *i*_3_ was chosen to correspond to *i*_Kr_. Furthermore, it was assumed that E-4031 acts to only alter the maximum conductance of *i*_Kr_ channels, without modulating their kinetics. Therefore during optimisation, only one pharmacological-specific parameter ${\overset{-}{g}}_{3}$, the maximum conductance of the *i*_Kr_(*i*_3_), was optimised to have a distinct value for each of the two datasets. All other model parameter values were shared between the datasets, since any AP waveshape variation was assumed to be due to the blocking effect of E-4031 on *i*_Kr_ alone. The optimised pSAN model was spontaneously active and AP fits to both datasets are shown in Figure 6, with RMS error between the model and corresponding data being 2.79 mV (control) and 3.91 mV (E-4031). Optimised parameter and initial values of model variables are given in Tables 7 and 8. Under control conditions, the optimised ${\overset{-}{g}}_{3}$ value was 2438.40 *μ*S/cm^2^ but was reduced to 1562.28 *μ*S·cm^−2^ under the action of E-4031. Model-generated membrane currents corresponding to these fits are shown in Figures 7(a) and 7(b). The amplitude of *i*_Kr_(*i*_3_) in the E-4031 model is evidently reduced from a mean peak value of 19.26 *μ*A/cm^2^ to 12.73 *μ*A/cm^2^, resulting in a prolongation of repolarization and a decreased maximum diastolic potential. Note that membrane currents *i*_1_, *i*_2_, *i*_4_, and *i*_*L*_, which are not directly affected by E-4031, still exhibit differences between the two simulations due to the voltage dependency of each current and its corresponding interaction with *i*_Kr_.

*(ii) Tissue-Specific Case. *Spatial heterogeneity in AP waveforms is evident throughout the atria, likely due to the differential expression of ion channels from sinoatrial node (SAN) to atrial regions [19]. APs were recorded from central sinoatrial node (cSAN) and right atrial (RA) tissue-intact myocytes in one rabbit SAN preparation (*n* = 1 for each cell type). For this case of tissue-specific optimisation, parameters for the maximum conductance $\left({\overset{-}{g}}_{j}\right)$ of each membrane current (an indicator of ion channel density) were set as data-specific parameters. Each cell type possessed distinct values for the ${\overset{-}{g}}_{j}$, while all remaining parameters were shared: the assumption being that ion channels with the same kinetic properties are present throughout the whole tissue. 

**Figure 8:**
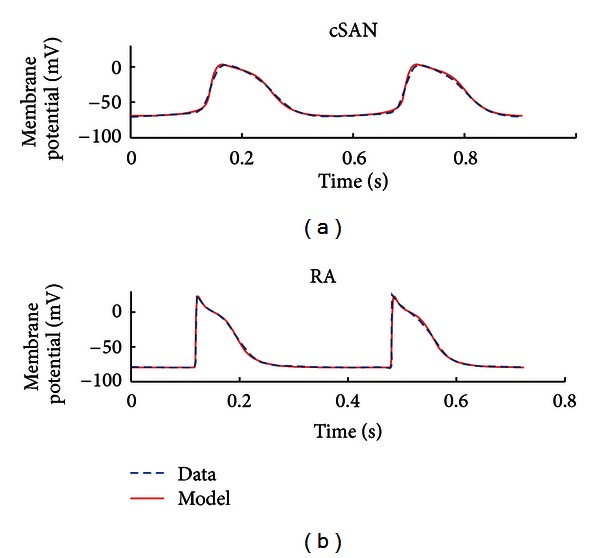
Tissue-specific multiobjective optimisation. Top and bottom panels illustrate the optimised model (solid traces) overlaid with experimental AP data (dashed traces), representing central sinoatrial node (cSAN) and right atrial (RA) APs recorded from the same tissue preparation.

**Figure 9:**
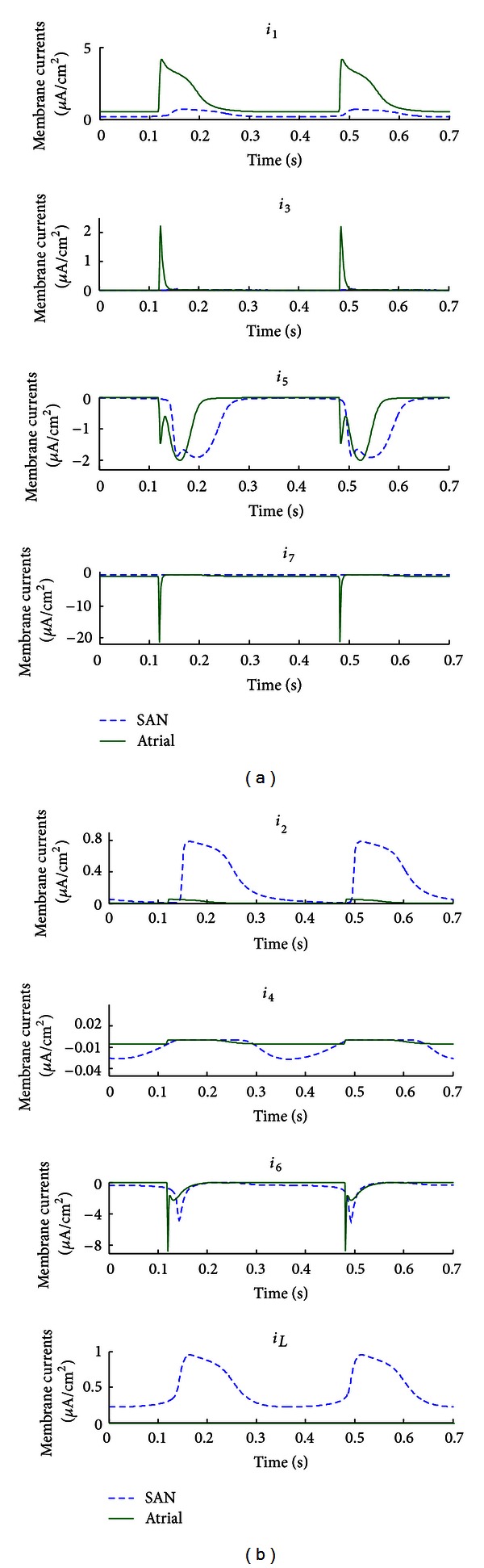
Model-generated membrane currents corresponding to the tissue-specific optimisation of Figure 8. Seven time-dependent currents and one leakage current were included in the model. All model parameters were shared between the two cell types, with the exception of the maximum membrane conductance for each of the ionic currents (i.e., ${\overset{-}{g}}_{1}\text{–}{\overset{-}{g}}_{7}$ and ${\overset{-}{g}}_{\text{L}}$, a total of eight parameters).

Figure 8 shows the fitted APs for both regions. The cSAN model was spontaneously active, whilst the atrial model was stimulated with rectangular pulses of 2 ms duration and 22 *μ*A/cm^2^ amplitude. A total of seven time-dependent currents and one leakage current were required to fit both datasets simultaneously. The RMS errors between the optimised models and corresponding data were 1.78 mV (cSAN) and 2.48 mV (RA). Values of all optimised parameters and initial variables are given in Tables 9 and 10. Significant channel density differences (maximum conductances) exist between cSAN and RA, which contribute to the difference in AP waveform. Comparison of cSAN and RA membrane currents is plotted in Figure 9, with a separate panel for each current from both cell types. Interestingly, ionic currents with the same kinetic parameters can display a very different time course for each cell type, due to the direct effect of the differences in ${\overset{-}{g}}_{j}$ and the indirect effects of voltage dependency in each current. 

## 4. Discussion

 In this study, a generic ionic model was optimised using a custom curvilinear gradient algorithm to fit a range of cardiac APs. The model was fitted to either single AP traces or simultaneously to multiple AP waveforms recorded from tissue-intact myocytes under different experimental conditions in rabbit SAN and/or atrial tissue preparations. AP waveforms could be well reproduced by the generic model, whose complexity lies intermediate between simple phenomenological formulations and biophysically detailed ionic models. Complex experimental data could be reproduced by the addition of extra ionic currents into the model. A major improvement over existing modelling approaches is that model parameters have been adjusted to accurately reproduce AP waveforms recorded under different pacing or pharmacological conditions from the same myocyte, reproducing complex AP characteristics while retaining physiologically realistic membrane current waveforms. 

Membrane current kinetics of the generic model were expressed in terms of two first-order gates (*p* and *q*). It is possible to incorporate more complex activation kinetics such as raising the gating variables to powers greater than one. Such a modification would, in principle, allow fits to sigmoidal time courses of activation during voltage clamps: a property of many membrane currents [22]. Surprisingly, our fits to multiple AP data did not require sigmoidal kinetics for the membrane currents, as would be the case if we were to reproduce voltage-clamp data. However if desired, voltage-clamp data could be included as an additional dataset to be fitted alongside the other AP records simultaneously. Even if the data required membrane currents to reproduce a sigmoidal onset of activation, this could still be achieved with the simplified kinetic structure of our genetic model. For example, we have fitted the Hodgkin and Huxley (HH) [5] *i*_K_ (*n* ^4^ kinetics) and *i*_Na_ (m^3^ h kinetics) membrane currents in response to a voltage step from −60 mV to +40 mV, using a total of two generic currents (for *i*_K_) and four generic currents (for *i*_Na_) respectively (not shown), indicating that even with highly simplified kinetics, the generic model is still able to reproduce a wide range of experimental data. The modeller can decide whether to amalgamate any generic currents obtained with our method into more complex formulations, as a first step towards formulating a more biophysically detailed model if desired.

Compared with previous studies using simplified cell formulations such as the Fitzhugh-Nagumo or Fenton-Cherry models [4, 14, 23], our generic model could accurately reproduce spontaneous AP waveforms recorded from cSAN, pSAN as well as paced AP waveforms from RA and LA myocytes. Although previously published phenomenological models were able to reproduce restitution curves and reasonable AP waveforms, we regard it necessary to reproduce accurate AP morphologies in modelling electrophysiological dynamics [3, 24, 25]. With our approach, a user-defined number of membrane currents can be defined, providing higher flexibility in reproducing even more complex electrophysiological dynamics. In order to retain the simplified nature of the model, additional ionic currents were included only when necessary (i.e., the target RMS error could not be achieved). Moreover, because of the similar formulation of each ionic current, many of these can be recombined if they are found to follow similar time course profiles during optimisation, facilitating the process of model reduction. It is important to note that prior to optimisation, we make no assumptions as to the ionic identity of each current, but allow the fitting process to determine the current density and kinetics based solely on the AP data. The only exception was for the drug-specific scenario (Figures 6 and 7), where, prior to optimisation, the maximum conductance of current *i*_3_ was chosen as the only parameter to be altered by E-4031, effectively preidentifying this current with *i*_Kr_ for this optimisation run only. We could equally have chosen any other conductance parameter in the model. Although *i*_3_ is preidentified with *i*_Kr_ in this case, *i*_3_ does not necessarily correspond to this membrane current for the other optimisations results of this study. The generic model approach outlined here provides a promising tool for tissue or whole-heart simulations due to its simplified nature and, therefore, computational efficiency. 

**Figure 10:**
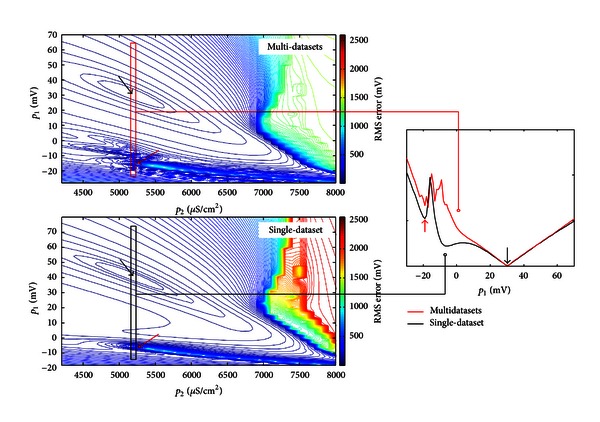
Normalised objective surface reproduced by the RMS error between model and experimental APs, against two optimised parameters *p*_1_ and *p*_2_ representing *i*_6_ reversal potential (*p*_1_) and *i*_5_ maximum membrane conductance (*p*_2_). Upper plot, 2D objective surface of multi-dataset based optimisation. Note the “noisy” local area indicated by the red arrow. The global minimum is labelled by the black arrow. Lower plot, 2D objective surface of single-dataset based optimisation. Inset: Zoomed 1D local objective surface near global minimum. When optimising the model to fit multiple datasets simultaneously, the number of local minima will be increased. However, each local minimum will be shallower compared to those of the single-dataset based surface.

Our multidataset-based optimisation results suggest that multiobjective experimental AP data can improve ionic model optimisation and predictive power of the model, provided the additional data includes information not present in the original dataset(s). Model optimisation using multiple data with similar AP characteristics, such as AP duration or amplitude, will tend to simply maintain the number and location of local minima on the objective surface. However, introducing additional datasets with extra information will smooth the objective surface by reducing the amplitude of any “surface ripple,” since each parameter point on the surface must now fit multiple data (see Figure 10). In addition, new datasets with distinct “information” will introduce more local minima onto the objective surface, making its topology more complex and thus confounding the search for the global optimum. We found that for multiple data optimisation, it was much more challenging to fit all datasets simultaneously, particularly if there were stringent constraints on parameters and parameter values were shared between the datasets. At the same time, the credibility of the ionic model is enhanced by its ability to simultaneously reproduce data obtained under variable experimental conditions

In addition, reconstructed membrane currents of the optimised models were found to follow physiologically realistic waveforms in SAN and atrial rabbit myocytes, consistent with current profiles predicted in existing biophysically detailed models [26, 27]. It is important to note that membrane currents with unrealistic time course profiles may perfectly reproduce a single AP record dataset, but these will generally fail to simultaneously reproduce multiple experimental data. Our results suggest that the multiobjective fitting approach can be used to accurately reconstruct underlying membrane current dynamics, since the additional information provided by the multiple data was important for their accurate reconstruction.

From the experimental APs recorded in response to pacing at uniform and random intervals, it can be seen that for both the PI = 200 ms and random paced datasets, there was some beat-beat variation in the time course of underlying membrane currents (Figure 5). At high pacing frequencies, a second AP is elicited shortly after the previous one, having a reduced AP duration and refractory period due to the reduction in magnitude of inward ionic currents. The generic model was optimised to simultaneously fit AP responses to regular and random pacing and could therefore reproduce this AP alternans at higher rates, which is important in understanding and simulating the pathophysiological mechanisms underlying reentrant activity and electrical remodelling in atrial fibrillation [28]. 

Many electrophysiological studies have employed dynamic AP clamp in the presence of selective ion channel blockers to visualise the time course of ionic currents underlying the AP. However, these methods suffer from the limitation that it is not currently possible to simultaneously visualise all membrane currents present in a given myocyte. An alternative approach may be to use integrative ionic model simulations which can reproduce cellular electrophysiological behaviour under multiple experimental conditions. The generic model of this study was optimised to fit multiple AP records recorded during control conditions and also under the influence of a selective ion channel blocker (E-4031). It was assumed that in the two conditions, all model parameters shared identical values, with the exception of the maximum membrane conductance of the blocked *i*_Kr_ current. We have found that fitting to a single AP dataset does not provide unique reconstructions of the time course of underlying membrane currents. Nevertheless, the membrane currents reconstructed using the multiobjective drug-specific optimisation of the present study reveal remarkably similar time course profiles to other existing biophysically detailed models. For example, rabbit SAN [29, 30] and rabbit atrial [26, 30] single cell ionic models display a similar time course to our *i*_Kr_, suggesting that this optimised current has been appropriately constrained by the drug-specific data optimisation. In contrast, some experimental AP-clamp data [31, 32] indicate a slower onset of *i*_Kr_, suggesting that we have not accurately isolated the time course of this current component, perhaps through inclusion of an *i*_to_ component. Additional datasets for use with multiobjective optimisation, such as, for example, voltage-clamp data in the presence of the drug, may further help in more accurately identifying the kinetics of this current.

The generic model approach outlined here could also be used to simulate the effects of antiarrhythmic drugs on whole heart or tissue simulations, due to the computational simplicity of the model. The approach could even be used with pharmacological agents known to have multiple effects, such as the partial block of more than one current. The user would only need to specify which model parameters are shared between datasets and which are drug-specific.

**Figure 11:**
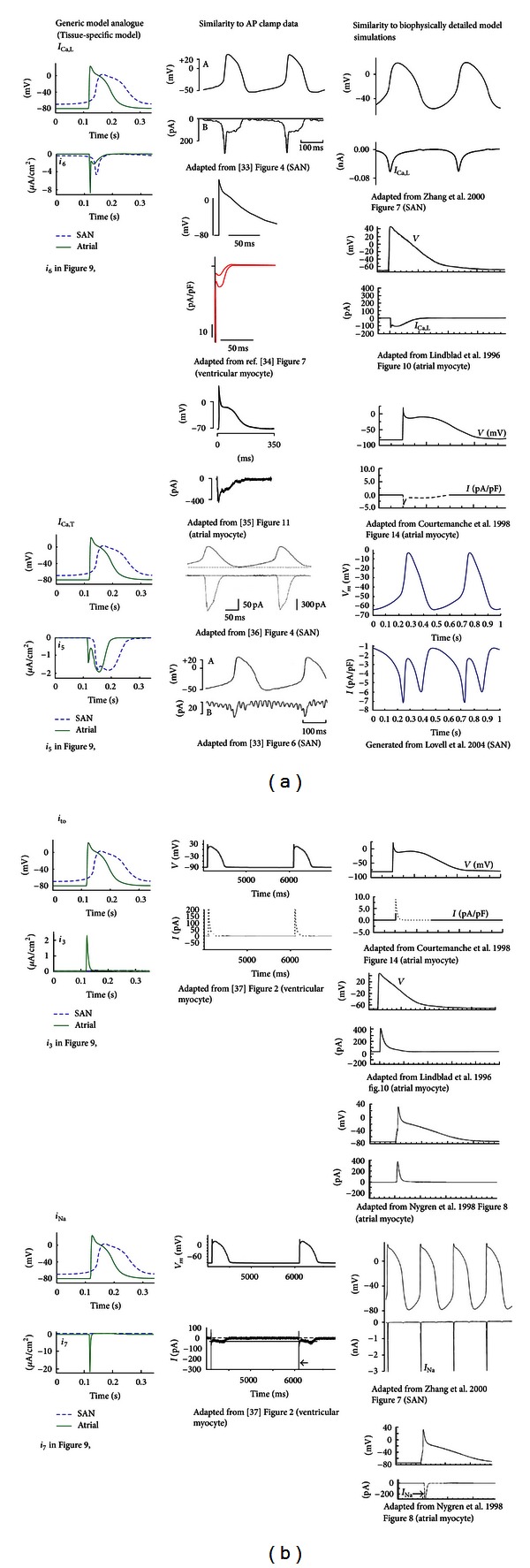
Comparison of the time course of generic model currents with experimental AP clamp data and biophysically-detailed model simulations.

The tissue-specific optimisation approach also allowed the generic model to be fitted to heterogeneous APs from different myocyte types in the same tissue preparation. It was assumed in this case that all model parameters were shared between datasets, except the maximum membrane conductance of each current. As was the case for the drug-specific optimisation, the reconstructed generic currents in the model resembled known profiles of ionic currents, based on their similarity to current profiles obtained from biophysically detailed models of cSAN and atrial rabbit myocytes, as well as published data of pharmacologically isolated ionic currents obtained using the dynamic AP clamp technique [33–38] (see Figure 11). In addition, comparison of the magnitudes of *i*_Na_ and *i*_to_ (*i*_7_ and *i*_3_, resp.) between SAN and atrial myocytes is in agreement with experimental data from isolated right atrial preparations [39, 40]. 

Furthermore, the excellent AP fits obtained for the tissue-specific case (Figure 8) indicate that this multiobjective optimisation approach could also provide an extension to previously published gradient models of cardiac tissue electrical activity, particularly models of SAN-atrial interaction. Compared with Lovell et al.'s work [29], all kinetic model parameters were shared by each dataset in this study; therefore any spatial regional differences are only reflected through variation in maximal membrane conductances. Compared to the gradient model published by Zhang et al. [30], the tissue-specific model of this study could accurately fit SAN and RA APs by optimising shared kinetic parameters. Compared with the work of Syed et al. [19] and Dastgheib et al. [18], who only optimised maximum ion channel conductance parameters [18, 19], all model parameters were included in the tissue-specific optimisation of Figure 8. We believe that estimating only conductance parameters while fixing ion channel kinetics parameters will excessively limit the parameter search space, reducing the accuracy of model fits, particularly when optimising against multiple datasets. 

Like most existing models, our generic model has certain limitations, representing compromises which are necessary to achieve simplified and computationally efficient descriptions of membrane current kinetics. While the model can accurately fit multiple AP data, we have not incorporated intracellular calcium cycling, changes in intracellular ion concentrations, metabolites, or ionic pumps and exchangers. These changes, if present, would impact the AP waveshape through our reconstructed membrane currents, which we assume to simply consist of two first-order voltage-dependent (*p* and *q*) gating processes. It is also important to note that optimisation of nonlinear models, particularly those with large numbers of parameters, may lead to nonunique parameter estimates. The generic model of this study is no exception. The symmetrical nature of the membrane current formulations indicates that the parameters of any two membrane currents can be interchanged to produce identical AP waveforms, due to the identical formulations for each current. A similar argument would hold for the *p*, *q* gating variables: their kinetic parameters can be interchanged within any current due to their symmetrical formulations. From such simple considerations, it can be concluded that parameters of the generic model cannot be uniquely determined, unless nonsymmetrical upper/lower bounds are imposed on individual parameters. However, we found the model currents can converge to similar waveforms, regardless of the initial parameter values used. These results indicate that the additional information provided by the multiple data was important for accurate reconstruction of membrane currents. In general, this was not possible when fitting the model to single datasets, even though the AP record itself could be well fitted [20, 41]. In other words, model behaviours, as opposed to parameters, tend to be well identified when extra experimental information is incorporated into the optimisation.

## 5. Conclusion

We have presented a generic model of cardiac electrical activity, capable of accurately reproducing action potential waveforms from multiple experimental data in any given myocyte. Furthermore, we have shown that our generic ionic model and multiobjective optimisation approach described in this study can provide an effective and efficient means to reconstruct the profiles of hidden ionic currents underlying the AP. Multiobjective fitting to multiple AP datasets appears to provide stringent constraints on the dynamics of underlying membrane currents, yielding reconstructed membrane current time course profiles in agreement with existing studies. The generic approach will allow the efficient computation of complex electrophysiological dynamics in whole heart simulations and will provide a valuable tool in elucidating the ionic mechanisms underlying cardiac electrical activity.

## Figures and Tables

**Table 1:** Parameter values for single AP dataset-based optimisation.

Parameter	Description	cSAN	pSAN	RA
${\overset{-}{g}}_{1}$	Maximum *I*_1_ conductance (*μ*S/cm^2^)	95.71	60.29	33.55
*E*_rev,1_	Reversal potential of *I*_1_ (mV)	−91.74	−92.80	−97.71
*k*_α*p*1_	Maximum value of α_*p*1_ (s^−1^)	4932.74	4990.73	3099.16
*s*_α*p*1_	Slope value for α_*p*1_ (mV^−1^)	−1.84	−2.92	−0.10
*k*_β*p*1_	Maximum value of β_*p*1_ (s^−1^)	15.26	41.15	4477.72
*s*_β*p*1_	Slope value for β_*p*1_ (mV^−1^)	2.48	5.00	0.15
*E*_αβ*p*1_	*E*_50_ value for α_*p*1_ and β_*p*1_ (mV)	−30.44	−62.41	−77.65
*k*_α*q*1_	Maximum value of α_*q*1_ (s^−1^)	1.40	6.61	170.9
*s*_α*q*1_	Slope value for α_*q*1_ (mV^−1^)	−3.86	−2.09	−0.01
*k*_β*q*1_	Maximum value of β_*q*1_ (s^−1^)	7.92	7.81	0.01
*s*_β*q*1_	Slope value for β_*q*1_ (mV^−1^)	0.10	0.92	0.19
*E*_αβ*q*1_	*E*_50_ value for α_*q*1_ and β_*q*1_(mV)	−36.17	−53.46	−80.46

${\overset{-}{g}}_{2}$	Maximum *I*_2_ conductance (*µ*S/cm^2^)	17722.1	17966.7	9962.91
*E*_rev,2_	Reversal potential of *I*_2_ (mV)	11.38	6.98	14.54
*k*_α*p*2_	Maximum value of α_*p*2_ (s^−1^)	0.44	2.80	300.89
*s*_α*p*2_	Slope value for α_*p*2_ (mV^−1^)	−4.98	−4.99	−0.20
*k*_β*p*2_	Maximum value of β_*p*2_ (s^−1^)	3851.95	4612.17	4030.31
*s*_β*p*2_	Slope value for β_*p*2_ (mV^−1^)	0.26	0.70	0.02
*E*_αβ*p*2_	*E*_50_ value for α_*p*2_ and β_*p*2_ (mV)	−47.98	−70.14	−51.73
*k*_α*q*2_	Maximum value of α_*q*2_ (s^−1^)	4.36	9.95	16.86
*s*_α*q*2_	Slope value for α_*q*2_ (mV^−1^)	3.51	3.04	0.20
*k*_β*q*2_	Maximum value of β_*q*2_ (s^−1^)	43.47	62.65	52.61
*s*_β*q*2_	Slope value for β_*q*2_ (mV^−1^)	−4.47	−4.67	−0.18
*E*_αβ*q*2_	*E*_50_ value for α_*q*2_ and β_*q*2_ (mV)	−36.24	−69.46	−75.74

${\overset{-}{g}}_{L}$	Maximum *I*_*L*_ conductance (*µ*S/cm^2^)	3.70	4.00	1.74
*E*_rev,*L*_	Reversal potential of *I*_*L*_ (mV)	−42.59	−69.32	−58.98

**Table 2:** Initial variable values for single AP dataset-based optimisation.

Variable	cSAN	pSAN	RA
*E*_*m*_ (mV)	−55.65	−74.09	−81.35
*p*_1_	0.23	0.063	0.79
*q*_1_	0.07	0.28	0.39
*p*_2_	0	0	0
*q*_2_	0.47	0.38	0.30

**Table 3:** Parameter values for LA multidataset-based optimisation.

Parameter	Description	Run 1	Run 2	Run 3
${\overset{-}{g}}_{1}$	Maximum *I*_1_ conductance (*µ*S · cm^−2^)	3044.83	1926.84	4547.51
*E*_rev,1_	Reversal potential of *I*_1_ (mV)	5.00*e* − 4	2.50*e* − 2	9.54*e* − 2
*k*_α*p*1_	Maximum value of α_*p*1_ (s^−1^)	4483.62	4425.83	4619.86
*s*_α*p*1_	Slope value for α_*p*1_ (mV^−1^)	−9.21*e* − 2	−7.72*e* − 2	−5.16*e* − 2
*k*_β*p*1_	Maximum value of β_*p*1_ (s^−1^)	0.21	11.60	20.29
*s*_β*p*1_	Slope value for β_*p*1_ (mV^−1^)	0.196	0.197	0.199
*E*_αβ*p*1_	*E*_50_ value for α_*p*1_ and β_*p*1_ (mV)	−28.97	−41.00	9.25
*k*_α*q*1_	Maximum value of α_*q*1_ (s^−1^)	1375.78	2117.98	1076.29
*s*_α*q*1_	Slope value for α_*q*1_ (mV^−1^)	−0.20	−0.194	−0.196
*k*_β*q*1_	Maximum value of β_*q*1_ (s^−1^)	4761.23	3987.49	4844.62
*s*_β*q*1_	Slope value for β_*q*1_ (mV^−1^)	3.20*e* − 3	8.00*e* − 4	2.80*e* − 3
*E*_αβ*q*1_	*E*_50_ value for α_*q*1_ and β_*q*1_ (mV)	21.57	27.50	15.69

${\overset{-}{g}}_{2}$	Maximum *I*_2_ conductance (*µ*S · cm^−2^)	42.24	29.84	66.49
*E*_rev,2_	Reversal potential of *I*_2_ (mV)	−80.00	−80.00	−80.14
*k*_α*p*2_	Maximum value of α_*p*2_ (s^−1^)	115.88	19.19	82.99
*s*_α*p*2_	Slope value for α_*p*2_ (mV^−1^)	−0.136	−0.117	−0.132
*k*_β*p*2_	Maximum value of β_*p*2_ (s^−1^)	217.08	164.94	283.28
*s*_β*p*2_	Slope value for β_*p*2_ (mV^−1^)	0.20	0.20	0.20
*E*_αβ*p*2_	*E*_50_ value for α_*p*2_ and β_*p*2_ (mV)	−65.89	−68.28	−60.91
*k*_α*q*2_	Maximum value of α_*q*2_ (s^−1^)	1210.40	985.06	1311.51
*s*_α*q*2_	Slope value for α_*q*2_ (mV^−1^)	−0.20	−0.193	−0.20
*k*_β*q*2_	Maximum value of β_*q*2_ (s^−1^)	2603.48	2516.13	3055.08
*s*_β*q*2_	Slope value for β_*q*2_ (mV^−1^)	4.00*e* − 4	1.15*e* − 2	1.20*e* − 3
*E*_αβ*q*2_	*E*_50_ value for α_*q*2_ and β_*q*2_ (mV)	−99.87	−97.95	−71.00

${\overset{-}{g}}_{3}$	Maximum *I*_3_ conductance (*µ*S · cm^−2^)	7192.04	4338.65	3904.66
*E*_rev,3_	Reversal potential of *I*_3_ (mV)	51.31	37.11	59.56
*k*_α*p*3_	Maximum value of α_*p*3_ (s^−1^)	64.45	129.11	79.20
*s*_α*p*3_	Slope value for α_*p*3_ (mV^−1^)	−0.09	−9.41*e* − 2	−0.104
*k*_β*p*3_	Maximum value of β_*p*3_ (s^−1^)	4309.81	4587.86	4598.48
*s*_β*p*3_	Slope value for β_*p*3_ (mV^−1^)	2.44*e* − 2	3.61*e* − 2	2.73*e* − 2
*E*_αβ*p*3_	*E*_50_ value for α_*p*3_ and β_*p*3_ (mV)	−39.76	−39.20	−39.52
*k*_α*q*3_	Maximum value of α_*q*3_ (s^−1^)	252.37	439.94	365.37
*s*_α*q*3_	Slope value for α_*q*3_ (mV^−1^)	9.78*e* − 2	0.118	0.10
*k*_β*q*3_	Maximum value of β_*q*3_ (s^−1^)	8.01	8.38	5.46
*s*_β*q*3_	Slope value for β_*q*3_ (mV^−1^)	−0.106	−0.073	−0.176
*E*_αβ*q*3_	*E*_50_ value for α_*q*3_ and β_*q*3_ (mV)	−96.83	−99.51	−95.50

${\overset{-}{g}}_{4}$	Maximum *I*_4_ conductance (*µ*S · cm^−2^)	39997.9	39857.1	38120.4
*E*_rev,4_	Reversal potential of *I*_4_ (mV)	72.48	62.36	75.24
*k*_α*p*4_	Maximum value of α_*p*4_ (s^−1^)	2392.17	2330.46	1884.15
*s*_α*p*4_	Slope value for α_*p*4_ (mV^−1^)	−0.159	−0.15	−0.161
*k*_β*p*4_	Maximum value of β_*p*4_ (s^−1^)	4999.04	4995.38	4938.09
*s*_β*p*4_	Slope value for β_*p*4_ (mV^−1^)	0.173	0.189	0.166
*E*_αβ*p*4_	*E*_50_ value for α_*p*4_ and β_*p*4_ (mV)	−48.04	−48.21	−48.04
*k*_α*q*4_	Maximum value of α_*q*4_ (s^−1^)	46.66	48.78	22.51
*s*_α*q*4_	Slope value for α_*q*4_ (mV^−1^)	0.132	0.125	0.124
*k*_β*q*4_	Maximum value of β_*q*4_ (s^−1^)	1309.81	1172.17	1677.50
*s*_β*q*4_	Slope value for β_*q*4_ (mV^−1^)	−0.18	−0.1.86	−0.16
*E*_αβ*q*4_	*E*_50_ value for α_*q*4_ and β_*q*4_ (mV)	−73.25	−74.12	−64.6

${\overset{-}{g}}_{5}$	Maximum *I*_5_ conductance (*µ*S · cm^−2^)	3577.89	4188.07	3353.90
*E*_rev,5_	Reversal potential of *I*_5_ (mV)	5.51	3.62	7.54
*k*_α*p*5_	Maximum value of α_*p*5_ (s^−1^)	1943.58	1303.25	1115.88
*s*_α*p*5_	Slope value for α_*p*5_ (mV^−1^)	−0.199	−0.20	−0.197
*k*_β*p*5_	Maximum value of β_*p*5_ (s^−1^)	42.81	57.75	38.07
*s*_β*p*5_	Slope value for β_*p*5_ (mV^−1^)	1.68*e* − 2	1.16*e* − 2	1.33*e* − 2
*E*_αβ*p*5_	*E*_50_ value for α_*p*5_ and β_*p*5_ (mV)	26.49	20.86	30.38
*k*_α*q*5_	Maximum value of α_*q*5_ (s^−1^)	433.93	668.38	497.48
*s*_α*q*5_	Slope value for α_*q*5_ (mV^−1^)	4.00*e* − 4	1.06*e* − 2	1.50*e* − 3
*k*_β*q*5_	Maximum value of β_*q*5_ (s^−1^)	1244.48	884.84	985.96
*s*_β*q*5_	Slope value for β_*q*5_ (mV^−1^)	−0.176	−9.46*e* − 2	−4.68*e* − 2
*E*_αβ*q*5_	*E*_50_ value for α_*q*5_ and β_*q*5_ (mV)	−98.74	−92.40	−97.38
${\overset{-}{g}}_{L}$	Maximum *I*_*L*_ conductance (*µ*S · cm^−2^)	107.47	123.02	92.50
*E*_rev,*L*_	Reversal potential of *I*_*L*_ (mV)	−99.98	−99.93	−99.89

**Table 4:** Initial model variable values for LA multidataset-based optimisation.

	Run 1	Run 2	Run 3
Variable (PI = 400 ms)			

*E*_*m*_ (mV)	−80.32	−79.26	−79.86
*p*_1_	0.99	0.95	0.70
*q*_1_	0	0	0
*p*_2_	0.07	2.70*e* − 2	2.20*e* − 2
*q*_2_	0.45	0.46	0.11
*p*_3_	6.00*e* − 4	0	3.00*e* − 4
*q*_3_	0.87	0.84	0.92
*p*_4_	2.70*e* − 3	4.40*e* − 3	2.2*e* − 3
*q*_4_	0.11	8.90*e* − 2	0.126
*p*_5_	4.00*e* − 3	0	2.00*e* − 4
*q*_5_	0.16	0.31	0.264

Variable (PI = 200 ms)	

*E*_*m*_ (mV)	−77.80	−77.80	−78.61
*p*_1_	0.99	0.99	0.72
*q*_1_	0	0	0
*p*_2_	0.90	0.90	2.70*e* − 2
*q*_2_	0.42	0.42	0.133
*p*_3_	1.80*e* − 3	1.80*e* − 3	4.00*e* − 4
*q*_3_	0.64	0.64	0.883
*p*_4_	6.90*e* − 3	6.90*e* − 3	2.70*e* − 3
*q*_4_	5.70*e* − 2	5.70*e* − 2	0.102
*p*_5_	8.10*e* − 3	8.10*e* − 3	1.50*e* − 3
*q*_5_	3.50*e* − 2	3.50*e* − 2	0.26

Variable (PI = 300 ms)	

*E*_*m*_ (mV)	−80.40	−82.14	−79.96
*p*_1_	0.99	0.52	0.69
*q*_1_	0	0	0
*p*_2_	6.60*e* − 2	4.50*e* − 2	2.20*e* − 2
*q*_2_	0.45	0.45	0.109
*p*_3_	6.00*e* − 3	5.00*e* − 4	3.00*e* − 4
*q*_3_	0.87	0.93	0.922
*p*_4_	2.60*e* − 3	2.90*e* − 3	2.20*e* − 3
*q*_4_	0.113	0.14	0.128
*p*_5_	2.00*e* − 4	6.00*e* − 4	1.00*e* − 4
*q*_5_	0.16	0.27	0.264

**Table 5:** Parameter values for random-paced LA optimisation.

Parameter	Description	*I*_1_	*I*_2_	*I*_3_	*I*_4_	*I*_5_	*I*_6_	*I*_7_	*I*_*L*_
$\overset{-}{g}$	Maximum conductance (*µ*S/cm^2^)	3791.62	1112.87	24.99	334.03	5211.15	615.19	39984.07	12.96
*E*_rev_	Reversal potential (mV)	−99.82	−90.46	−60.73	−27.89	53.14	30.14	73.64	−82.72
*k*_α*p*_	Maximum value of α_*p*_ (s^−1^)	4680.75	2452.15	4309.86	1351.75	20.51	3406.11	1547.59	
*s*_α*p*_	Slope value for α_*p*_ (mV^−1^)	−7.40*e* − 2	−0.19	−0.16	−0.20	−8.40*e* − 2	−0.19	−0.19	
*k*_β*p*_	Maximum value of β_*p*_ (s^−1^)	1577.04	17.13	1882.61	97.24	1035.90	4991.79	4097.16	
*s*_β*p*_	Slope value for β_*p*_ (mV^−1^)	0.17	3.6*e* − 2	0	2.10*e* − 4	1.8*e* − 4	7.40*e* − 2	0.15	
*E*_αβ*p*_	*E*_50_ value for α_*p*_ and β_*p*_ (mV)	−99.90	22.84	14.65	−64.50	7.32	−43.38	−72.40	
*k*_α*q*_	Maximum value of α_*q*_ (s^−1^)	8.78	133.46	201.88	1342.22	967.19	230.35	4.50	
*s*_α*q*_	Slope value for α_*q*_ (mV^−1^)	0	1.20*e* − 3	0.19	0.20	0.20	0.17	0.19	
*k*_β*q*_	Maximum value of β_*q*_ (s^−1^)	4976.46	1285.70	3.7*e* − 3	2100.13	145.50	19.01	1159.70	
*s*_β*q*_	Slope value for β_*q*_ (mV^−1^)	0	−0.11	−3.1*e* − 2	−0.16	−3.40*e* − 2	−0.19	−0.19	
*E*_αβ*q*_	*E*_50_ value for α_*q*_ and β_*q*_ (mV)	−67.02	−94.50	−21.182	−100	0.32	−100	−99.88	

**Table 6:** Initial variable values for random-paced optimisation.

Variable	400 ms	200 ms	Random
*E*_*m*_ (mV)	−78.79	−77.96	−77.96
*p*_1_	0.99	0.99	0.99
*q*_1_	0.004	0.0014	0.0014
*p*_2_	0.000008	0.13	0.13
*q*_2_	0.0430	0.074	0.074
*p*_3_	0	0	0
*q*_3_	1	1	1
*p*_4_	0.82	0.74	0.74
*q*_4_	0.00940	0.0073	0.0073
*p*_5_	0.00009	0.000031	0.000031
*q*_5_	0.98	0.99	0.99
*p*_6_	0.00056	0.00094	0.00094
*q*_6_	0.32	0.1	0.1
*p*_7_	0.18	0.17	0.17
*q*_7_	0.00035	0.000095	0.000095

**Table 7:** Parameter values for drug-specific optimisation.

Parameter	Description	*I*_1_	*I*_2_	*I*_3_	*I*_4_	*I*_*L*_
$\overset{-}{g}$	Maximum conductance (*µ*S/cm^2^)	97	127.61	2438.40 (Control)	12279.67	13.58
				1562.28 (E-4031)		
*E*_rev_	Reversal potential (mV)	−55.54	−97.79	−79.96	43.39	−99.56
*k*_α*p*_	Maximum value of α_*p*_ (s^−1^)	4988.66	3178.26	3128	2545.56	
*s*_α*p*_	Slope value for α_*p*_ (mV^−1^)	−8.40*e* − 2	−6.80*e* − 3	−0.2	−9.386*e* − 2	
*k*_β*p*_	Maximum value of β_*p*_ (s^−1^)	2383.24	4828.39	72.52	1965.64	
*s*_β*p*_	Slope value for β_*p*_ (mV^−1^)	0.19	1.70*e* − 3	1.14*e* − 2	2.40*e* − 4	
*E*_αβ*p*_	*E*_50_ value for α_*p*_ and β_*p*_ (mV)	−89.62	16.61	9.81	7.38	
*k*_α*q*_	Maximum value of α_*q*_ (s^−1^)	587.81	85.64	162.53	0.83	
*s*_α*q*_	Slope value for α_*q*_ (mV^−1^)	−0.1	−1.50*e* − 5	2.54*e* − 2	0.2	
*k*_β*q*_	Maximum value of β_*q*_ (s^−1^)	4049.79	920.57	185.48	12.01	
*s*_β*q*_	Slope value for β_*q*_ (mV^−1^)	1.90*e* − 2	0.184	−0.14	−7*e* − 4	
*E*_αβ*q*_	*E*_50_ value for α_*q*_ and β_*q*_ (mV)	−84.76	−17.22	−99.92	−22.4	

**Table 8:** Initial variable values for drug-specific optimisation.

Variable	Control	E-4031
*E*_*m*_ (mV)	−76.90	−68.10
*p*_1_	0.94	0.98
*q*_1_	0.17	0.14
*p*_2_	0.37	0.37
*q*_2_	0.03	0
*p*_3_	0.04	0
*q*_3_	0.22	0.03
*p*_4_	0	0.004
*q*_4_	0.08	0.085

**Table 9:** Parameter values for tissue-specific optimisation.

Parameter	Description	*I*_1_	*I*_2_	*I*_3_	*I*_4_	*I*_5_	*I*_6_	*I*_7_	*I*_*L*_
$\overset{-}{g}$	Maximum conductance (*µ*S/cm^2^)	1577.21	1154.71	24.99	1014.53	2469.15	1376.76	15.83	10.08
	cSAN (upper)/RA (lower)	7526.58	66.55	76.99	50.00	2591.45	370.33	39997.86	4.30*e* − 2
*E*_rev_	Reversal potential (mV)	−99.65	−97.08	−60.94	−29.09	63.89	30.66	80.96	−91.40
*k*_α*p*_	Maximum value of α_*p*_ (s^−1^)	4843.48	2636.70	4265.53	1197.10	25.37	3472.10	2083.24	
*s*_α*p*_	Slope value for α_*p*_ (mV^−1^)	−9.09*e* − 2	−0.168	−0.131	−0.1.99	−8.54*e* − 2	−18.05*e* − 2	−0.196	
*k*_β*p*_	Maximum value of β_*p*_ (s^−1^)	2227.67	18.19	1505.24	138.93	1448.56	4999.96	4601.00	
*s*_β*p*_	Slope value for β_*p*_ (mV^−1^)	0.175	2.65*e* − 2	2.76*e* − 4	0.21*e* − 3	7.27*e* − 5	8.52*e* − 2	0.158	
*E*_αβ*p*_	*E*_50_ value for α_*p*_ and β_*p*_ (mV)	−96.10	−1.19	23.46	−61.64	−0.127	−43.024	−56.47	
*k*_α*q*_	Maximum value of α_*q*_ (s^−1^)	19.74	19.48	357.63	1068.00	1118.86	620.38	78.65	
*s*_α*q*_	Slope value for α_*q*_ (mV^−1^)	−4.23*e* − 3	2.6*e* − 3	0.198	0.20	0.20	18.47*e* − 2	0.195	
*k*_β*q*_	Maximum value of β_*q*_ (s^−1^)	4974.17	1236.21	209.24	2699.34	303.27	64.29	455.47	
*s*_β*q*_	Slope value for β_*q*_ (mV^−1^)	1.13*e* − 4	−0.117	−2.55*e* − 2	−0.177	−1.99*e* − 2	−0.187	−0.181	
*E*_αβ*q*_	*E*_50_ value for α_*q*_ and β_*q*_ (mV)	−42.60	−98.56	−36.83	−100.00	−4.22	−83.15	−92.11	

**Table 10:** Initial variable values for tissue-specific optimisation.

Variable	cSAN	RA
*E*_*m*_ (mV)	−69.08	−79.93
*p*_1_	0.996	0.97
*q*_1_	0.0036	0.0035
*p*_2_	0.208	0.093
*q*_2_	0.0088	0.009
*p*_3_	0	0
*q*_3_	0.847	0.87
*p*_4_	0.77	0.32
*q*_4_	0.0008	0.0072
*p*_5_	0.0001	0.000032
*q*_5_	0.946	0.95
*p*_6_	0.0068	0.0009
*q*_6_	0.398	0.84
*p*_7_	0.0366	0.0047
*q*_7_	0.0021	0.015

## References

[1] Sigg DC, Iaizzo PA, Xiao YF, He B (2010). *Cardiac Electrophysiology Methods and Models*.

[2] Winslow RL, Scollan DF, Holmes A, Yung CK, Zhang J, Jafri MS (2000). Electrophysiological modeling of cardiac ventricular function: from cell to organ. *Annual Review of Biomedical Engineering*.

[3] Fink M, Niederer SA, Cherry EM (2011). Cardiac cell modelling: observations from the heart of the cardiac physiome project. *Progress in Biophysics and Molecular Biology*.

[4] Fitzhugh R (1961). Impulses and physiological states in theoretical models of nerve membrane. *Biophysical Journal*.

[5] Hodgkin AL, Huxley AF (1952). A quantitative description of membrane current and its application to conduction and excitation in nerve. *The Journal of Physiology*.

[6] Courtemanche M, Skaggs W, Winfree AT (1990). Stable three-dimensional action potential circulation in the Fitzhugh-Nagumo model. *Physica D*.

[7] Kogan BY, Karplus WJ, Billett BS, Pang AT, Karagueuzian HS, Khan SS (1991). The simplified FitzHugh-Nagumo model with action potential duration restitution: effects on 2D wave propagation. *Physica D*.

[8] Aliev RR, Panfilov AV (1996). A simple two-variable model of cardiac excitation. *Chaos, Solitons and Fractals*.

[9] Nash MP, Panfilov AV (2004). Electromechanical model of excitable tissue to study reentrant cardiac arrhythmias. *Progress in Biophysics and Molecular Biology*.

[10] Luo CH, Rudy Y (1991). A model of the ventricular cardiac action potential. Depolarization, repolarization, and their interaction. *Circulation Research*.

[11] Beeler GW, Reuter H (1977). Reconstruction of the action potential of ventricular myocardial fibres. *The Journal of Physiology*.

[12] Fenton FH, Karma A (1998). Vortex dynamics in three-dimensional continuous myocardium with fiber rotation: filament instability and fibrillation. *Chaos*.

[13] Fenton FH, Cherry EM, Hastings HM, Evans SJ (2002). Multiple mechanisms of spiral wave breakup in a model of cardiac electrical activity. *Chaos*.

[14] Cherry EM, Ehrlich JR, Nattel S, Fenton FH (2007). Pulmonary vein reentry—properties and size matter: insights from a computational analysis. *Heart Rhythm*.

[15] Nattel S, Burstein B, Dobrev D (2008). Atrial remodeling and atrial fibrillation: mechanisms and implications. *Circulation*.

[16] Courtemanche M, Ramirez RJ, Nattel S (1998). Ionic mechanisms underlying human atrial action potential properties: insights from a mathematical model. *The American Journal of Physiology*.

[17] Cherry EM, Fenton FH (2007). A tale of two dogs: analyzing two models of canine ventricular electrophysiology. *The American Journal of Physiology*.

[18] Dastgheib ZS, Azemi A, Khademi M (2009). Identification of ionic conductances in a reentry model of ventricular myocardium cells. *Journal of Applied Sciences*.

[19] Syed Z, Vigmond E, Nattel S, Leon LJ (2005). Atrial cell action potential parameter fitting using genetic algorithms. *Medical and Biological Engineering and Computing*.

[20] Dokos S, Lovell NH (2004). Parameter estimation in cardiac ionic models. *Progress in Biophysics and Molecular Biology*.

[21] Kodama I, Boyett MR, Nikmaram MR, Yamamoto M, Honjo H, Niwa R (1999). Regional differences in effects of E-4031 within the sinoatrial node. *The American Journal of Physiology*.

[22] Hille B (2001). *Ion Channels of Excitable Membranes*.

[23] Bueno-Orovio A, Cherry EM, Fenton FH (2008). Minimal model for human ventricular action potentials in tissue. *Journal of Theoretical Biology*.

[24] Cherry EM, Fenton FH (2004). Suppression of alternans and conduction blocks despite steep APD restitution: electrotonic, memory, and conduction velocity restitution effects. *The American Journal of Physiology*.

[25] Tusscher KHWJT, Panfilov AV (2006). Alternans and spiral breakup in a human ventricular tissue model. *The American Journal of Physiology*.

[26] Lindblad DS, Murphey CR, Clark JW, Giles WR (1996). A model of the action potential and underlying membrane currents in a rabbit atrial cell. *The American Journal of Physiology*.

[27] Dokos S, Celler B, Lovell N (1996). Ion currents underlying sinoatrial node pacemaker activity: a new single cell mathematical model. *Journal of Theoretical Biology*.

[28] Nattel S (2002). New ideas about atrial fibrillation 50 years on. *Nature*.

[29] Lovell NH, Cloherty SL, Celler BG, Dokos S (2004). A gradient model of cardiac pacemaker myocytes. *Progress in Biophysics and Molecular Biology*.

[30] Zhang H, Holden AV, Kodama I (2000). Mathematical models of action potentials in the periphery and center of the rabbit sinoatrial node. *The American Journal of Physiology*.

[31] Faber GM, Silva J, Livshitz L, Rudy Y (2007). Kinetic properties of the cardiac L-type Ca^2+^ channel and its role in myocyte electrophysiology: a theoretical investigation. *Biophysical Journal*.

[32] Hancox JC, Levi AJ, Witchel HJ (1998). Time course and voltage dependence of expressed HERG current compared with native “rapid” delayed rectifier K current during the cardiac ventricular action potential. *Pflugers Archiv—European Journal of Physiology*.

[33] Doerr T, Denger R, Trautwein W (1989). Calcium currents in single SA nodal cells of the rabbit heart studied with action potential clamp. *Pflugers Archiv—European Journal of Physiology*.

[34] Kim N, Cannell MB, Hunter PJ (2010). Changes in the calcium current among different transmural regions contributes to action potential heterogeneity in rat heart. *Progress in Biophysics and Molecular Biology*.

[35] Yue L, Feng J, Gaspo R, Li GR, Wang Z, Nattel S (1997). Ionic remodeling underlying action potential changes in a canine model of atrial fibrillation. *Circulation Research*.

[36] Mangoni ME, Couette B, Marger L, Bourinet E, Striessnig J, Nargeot J (2006). Voltage-dependent calcium channels and cardiac pacemaker activity: from ionic currents to genes. *Progress in Biophysics and Molecular Biology*.

[37] Berecki G, Zegers JG, Wilders R, van Ginneken AC (2007). Cardiac channelopathies studied with the dynamic action potential-clamp technique. *Methods in Molecular Biology*.

[38] Nygren A, Fiset C, Firek L (1998). Mathematical model of an adult human atrial cell: the role of K^+^ currents in repolarization. *Circulation Research*.

[39] Honjo H, Boyett MR, Kodama I, Toyama J (1996). Correlation between electrical activity and the size of rabbit sino-atrial node cells. *The Journal of Physiology*.

[40] Lei M, Boyett MR (1998). Inhibition of transient outward current, it(to), by flecainide and quinidine in rabbit isolated sinoatrial node cells. *The Journal of Physiology*.

[41] Sarkar AX, Sobie EA (2010). Regression analysis for constraining free parameters in electrophysiological models of cardiac cells. *PLoS Computational Biology*.

